# Selected Extracellular microRNA as Potential Biomarkers of Multiple Sclerosis Activity—Preliminary Study

**DOI:** 10.1007/s12031-014-0476-3

**Published:** 2014-12-10

**Authors:** Magdalena Justyna Kacperska, Karol Jastrzebski, Bartlomiej Tomasik, Jakub Walenczak, Maria Konarska-Krol, Andrzej Glabinski

**Affiliations:** 1 Department of Neurology and Stroke, Medical University of Lodz, Zeromskiego 113, 90-549 Lodz, Poland; 2 Department of Pediatrics, Oncology, Hematology and Diabetology, Medical University of Lodz, Sporna 36/50, 91-738 Lodz, Poland; 3 Medical University of Lodz, Kosciuszki 4, 90-419 Lodz, Poland; 4 Department of Propedeutics of Neurology with Stroke Unit, Medical University of Lodz, Kopernik Hospital in Lodz, Pabianicka 62, 93-513 Lodz, Poland

**Keywords:** Multiple sclerosis, Central nervous system, Biomarker, Activation of the immune system, miRNA-let7a, miRNA-92a, miRNA-648a, EDSS, Plasma, Expression analysis

## Abstract

Multiple sclerosis (MS) is an autoimmune demyelinating disease of the central nervous system (CNS). Four distinct disease courses are known, although approximately 90 % of patients are diagnosed with the relapsing-remitting form (RRMS). The name “multiple sclerosis” pertains to the underlying pathology: the presence of demyelinating plaques in the CNS, in particular in the periventricular region, corpus callosum, cervical spine, and the cerebellum. There are ongoing efforts to discover biomarkers that would allow for an unequivocal diagnosis, assess the activity of inflammatory and neurodegenerative processes, or warn of disease progression. At present, small noncoding RNA particles-microRNA (miRNA, miR) seem to be particularly noteworthy, as they take part in posttranscriptional regulation of expression of various genes. Changes in composition as well as function of miRNA found in body fluids of MS patients are subjects of research, in the hope they prove accurate markers of MS activity. This preliminary study aims to evaluate the expression of selected extracellular microRNA particles (miRNA-let-7a, miRNA-92a, miRNA-684a) in patients experiencing MS relapse and remission, with healthy volunteers serving as a control group and to evaluate the correlation between miRNA expression and selected clinical parameters of those patients. Thirty-seven patients suffering from MS formed two examined groups: 20 patients undergoing relapse and 17 in remission. Thirty healthy volunteers formed the control group. All patients who were subjects to peripheral blood sampling had been hospitalized in the Department of Neurology and Stroke^1^. Four milliliters of venous whole blood had been collected into EDTA tubes. The basis for the selection of the three particular miRNA investigated in this study (miRNA-let-7a, miRNA-92a, miRNA-684a) was a preliminary bioinformatic analysis of data compiled from several medical databases, including Ovid MEDLINE®, Embase, Cochrane Database of Systematic Reviews (CDSR), miRWalk, and miRBase. The isolation of extracellular microRNA from plasma was carried out using miRNeasy Mini Kit (Qiagen) reagents. The reverse transcription was carried out with TaqMan® MicroRNA Reverse Transcription Kit (Applied Biosystems), as per manufacturers’ instructions. Standard microRNA TaqMan® tests (Applied Biosystems) were used for miRNA quantification. The qPCR were performed on a 7900 HT Fast Real-Time PCR System (Applied Biosystems) and analyzed using Sequence Detection System 2.3 software. In addition, all patients at the Department of Neurology and Stroke undergo a routine complete blood count with differential. The main objective of this study was to evaluate the expression of selected microRNA (has-miR-let-7a, miR-92a, and miR-648a) in the plasma of patients with MS during a relapse as well as in remission and attempt to correlate the acquired data with clinically relevant parameters of the disease. Finding such correlations may potentially lead to the use of miRNA as a biomarker of MS, which could help diagnose the disease and assess its severity and the efficacy of treatment. The difference in the expression of has-miR-let-7a in the remission group and the control group was statistically significant (*p* = 0.002). Similarly, the expression of miRNA-648a in patients in remission was significantly different from the expression in the control group (*p* = 0.02). Analysis of the correlation between the expression of miRNA-92a and the severity of the disease as measured by the EDSS scale in patients undergoing relapse showed significant negative linear correlation (*r* = −0.54, *p* = 0.01). Higher miR-648a expression correlated with more frequent flare-ups in the joint group of patients in remission and relapse (*p* = 0.03). This study is one of the few that demonstrate significantly changed expression of selected extracellular miRNA in plasma of MS patients and correlate those findings with clinical parameters. These observations may suggest that some miRNA subsets may be potential biomarkers for MS activity.

## Introduction

Multiple sclerosis (MS) is a chronic inflammatory-demyelinating disease of the central nervous system (CNS), characterized by focal damage to oligodendrocytes, axonal and neuronal degeneration, and typical demyelinating plaque formation in the CNS. The name “multiple sclerosis” pertains to the underlying pathology: the presence of demyelinating plaques in the CNS, in particular in the periventricular region, corpus callosum, cervical spine, and the cerebellum. The prevalence in Poland is 40–60 per 100,000 people (Kulakowska et al. 2010). The disease’s onset is typically between 20 and 40 years. Women are affected twice as often as men. Lublin and Reingold classification describes four disease courses: relapsing-remitting MS (RRMS), primary-progressive MS (PPMS), secondary-progressive MS (SPMS), and progressive-relapsing MS (Lublin and Reingold 1996).

Symptoms of MS are manifold and are a result of damage to various parts of the central nervous system, from the spinal cord to the brain cortex. In 85 to 90 % of patients, the disease first manifests as a clinically isolated syndrome (CIS). This term describes the first episode of neurologic symptoms caused by inflammatory or demyelinating lesions in the CNS (Kappos et al. 2006; Optic Neuritis Study Group 1997). Spastic paresis of upper and lower limbs is a typical symptom and may manifest only during exacerbations or between them if the recovery of function is not complete. Approximately 70 % of patients experience some degree of spasticity, most commonly of the lower limbs (Barnes et al. 2003). Brain stem and cerebellar symptoms include dizziness and vertigo, dysarthria, nystagmus, ataxia, gait instability, dysmetria, and dysdiadochokinesia. Bladder dysfunction occurs in around 75 % of MS patients. Patients experience urinary retention, incontinence, or a combination of both of the above (Del Popolo et al. 2008). Cognitive impairment and mental disorders including but not limited to clinical depression, bipolar disorder, short-term memory loss, attention deficit, verbal fluency deficit, poor visual-spatial ability, and chronic fatigue syndrome are found in over 65 % of patients (Krupp et al. 1995). The etiology remains unknown. The disease is most likely caused by complex interactions between environmental and genetic factors, which lead to an impaired immune response, resulting in the destruction of myelin sheaths, oligodendrocytes, axons, and neurons, as well as the formation of characteristic areas of demyelination (plaques) in the central nervous system (Sospedra and Martin 2005). Experimental autoimmune encephalomyelitis (EAE), induced by immunization with myelin antigens, provides an animal model for multiple sclerosis. EAE research proves that demyelinating lesions in the CNS are a result of autoaggression. Recent discoveries indicate that IL-17 producing Th lymphocytes (Th17) play a special role in the pathogenesis of autoimmune disorders such as MS and EAE (Bartel 2004).

Though multiple sclerosis (MS) is a chronic inflammatory-demyelinating disease of the CNS of considerable prevalence in the Polish population, featuring a varied symptomatology and unclear etiology, research on the cellular and molecular level is constantly progressing towards a better understanding of the disorder.

## Extracellular miRNA as Biomarkers of Multiple Sclerosis

There are ongoing efforts to discover biomarkers that would allow for fast and accurate diagnosis, prophylaxis, monitoring of the activity of inflammatory and neurodegenerative processes as well as the degree of activation of patient’s immune system. At present, a group of endogenous, single-stranded noncoding RNA, or miRNA, draws researchers’ close attention. miRNA constitute the largest group of short regulatory RNA particles. They were first described in nematodes, then in fruit flies, fish and mice (Alvarez-Garcia and Miska 2005; Williams 2008). In mammals, miRNA play an enormous role in both physiological and pathological processes, such as cell differentiation, angiogenesis, apoptosis, inflammation, neurological disorders, and bacterial and viral infections, among others (Alvarez-Garcia and Miska 2005; Denli et al. 2004; Williams 2008). miRNAs are processed from precursor molecules (pri-miRNAs) which could be transcribed by RNA polymerase II from their own independent genes or derived from introns of protein-coding genes. In the case of the former, the primary transcript is processed with the Drosha enzyme into 60–80 nucleotide-long miRNA precursors (pre-miRNAs). The Drosha type-III RNase generates a double stranded RNA with a phosphate group at the 5′-end and a hydroxyl group and two unpaired nucleotides at the 3′-end (Bohnsack et al. 2004). In the latter case, splicing and debranching of very short introns (mirtrons) occurrs and pre-miRNAs are produced without the Drosha step (Krol et al. 2010). After initial processing in the nucleus, the 20–22 nucleotide-long pre-miRNA are then exported to the cytoplasm by the complex of Exportin 5 (Exp5) and Ran-GTP (Bohnsack et al. 2004; Schwarz et al. 2003). A Dicer enzyme cleaves the molecules, which then form RNA-induced silencing complexes (RISC) (Tomari et al. 2004). By attaching themselves to complementary sequences of the target RNA, the RISC complexes improve their stability and help in mRNA translation. miRNA inhibits protein synthesis by interacting with partially complementary regions near the 3′-end, which do not undergo translation. microRNA controls the expression of many genes, but it merits attention that particular miRNA molecules are capable of regulating specific mRNA. Conducted studies indicate the important role of small noncoding miRNA as growth, differentiation, and apoptosis regulators for selected cell lines of the immune system (Gandhi et al. 2013). This is achieved by affecting transcription factors, proapoptotic proteins, and signal transduction pathways (Gandhi et al. 2013). A positive correlation has been shown between changes in miRNA expression and the development of autoimmunity (Lehmann et al. 2012). Perhaps most importantly, miRNA abnormalities can be studied by assessing changes in freely circulating extracellular miRNA (Lehmann et al. 2012).

## Materials and Methods

### Patients

Thirty-seven patients suffering from MS agreed to take part in the study, with 20 patients undergoing a relapse without treatment (19 women and 1 man) and 17 in remission without treatment (15 women and 2 men). Thirty healthy volunteers (18 women and 12 men) formed the control group. Detailed patients’ histories were taken, especially concerning MS, and included early symptoms, year of diagnosis, presentation in the first year upon diagnosis, number of relapses and hospitalizations, management, familial antecedents, and comorbidities. Neurological exams were performed to assess patients using the Expanded Disability Status Scale (EDSS).

### The Isolation of Blood Plasma

Four milliliters of venous blood were collected at a time and put into EDTA tubes. After centrifugation was undertaken to obtain plasma, all biologic material was stored at −80 °C to await analysis. The blood was centrifuged (5000 rpm, 20 min, 23 °C), and later the top layer containing plasma was carefully moved into test tubes (2 × 2 ml). The tubes of plasma labeled with patient’s individual identification numbers were then stored at −80 °C on the premises of the Department of Neurology and Stroke Unit. Actual analyses took place in the Central Laboratory of the Medical University of Lodz.

### miRNA Selection

A bioinformatic analysis identified three extracellular miRNA molecules as good potential candidates for markers of MS (miRNA-let-7a, miRNA-92a, miRNA-684a). They correlated well with MS progression as well as markers of inflammation, such as IL-17 and IL-7, among others, while IL-7 is also implicated in the pathogenesis of MS. The following databases were used: Ovid MEDLINE®, Embase, Cochrane Database of Systematic Reviews (CDSR), miRWalk, and miRBase (Griffiths-Jones et al. 2008; Dweep et al. 2011).

### miRNA Expression Analysis

The isolation of extracellular microRNA from plasma was carried out using miRNeasy Mini Kit (Qiagen) reagents. A 0.2-ml plasma was added to 1 ml of QIAzol reagent, shaken vigorously, and incubated for 5 min at room temperature to promote a complete dissociation of nucleoprotein complexes. Subsequently, 25 fmols of *Caenorhabditis elegans* miRNA-39 mimic (cel-miRNA-39) were added to serve as an exogenous, “spiked-in” control of the purification efficiency. After adding 320 μl of chloroform to separate the aqueous and phenolic phases, the homogenate was vigorously shaken for 45 s and stored for 5 min at room temperature. After centrifuging at 14,000*g* for 20 min, total RNA was precipitated from the aqueous phase using 100 % ethanol. The purification of total RNA was achieved using QuiagenmiRNeasy spin columns according to the protocol provided by the manufacturer. The RNA was then eluted from the column by adding 30 μl of RNase-free water.

The reverse transcription of 5 μl of the eluate in a 15-μl reaction was carried out using a TaqMan® MicroRNA Reverse Transcription Kit (Applied Biosystems), as per the manufacturers’ instructions. The standard MicroRNA TaqMan® tests (Applied Biosystems) used for miRNA quantification were the following: HSA-let-7a (test ID: 000377), HSA-miR-92a (test ID: 000431), HSA-miR-648A (test ID: 001601), as well as cel-miR-39 (test ID: 000200) as control. The 20-μl reaction mix required for the quantitative PCR contained 1.33 μl of the product from the RT reaction, 10 μl of TaqMan Universal PCR Master Mix, 1 μl of TaqMan miRNA assay (20×), and nuclease-free water. Reaction plates were incubated in a 96-well thermal cycling plate at 95 °C for 10 min and then underwent 40 cycles of 15 s at 95 °C and 1 min at 60 °C. All reactions were performed in duplicate.

The qPCR were performed on a 7900 HT Fast Real-Time PCR System (Applied Biosystems) and analyzed using Sequence Detection System 2.3 software. Relative quantitation (RQ) was calculated using the 2^−ΔCt^ method, where ΔCt symbolized the difference of Ct between sample and reference miRNA.

### Blood Count Analyses

All 37 patients participating in the study also underwent a complete blood count, as part of the routine diagnostic panel for every MS patient at the Central Veterans’ Teaching Hospital No. 2. Whole blood was collected into tubes containing an anticoagulant (EDTA) by the nursing staff of the Department of Neurology and Stroke in Lodz per doctor’s request and analyzed in the hospital lab. The specific equipment used was Siemens ADVIA 2120i, according to the manufacturer’s protocol.

### Statistical Analysis

Nominal variables were given as numbers with appropriate percentage whereas continuous variables as means with standard deviation. The normality of distribution was verified using the Shapiro-Wilk *W* test. Grubbs’ test was used to detect outliers. Differences between both groups were analyzed using the nonparametric Mann-Whitney *U* test. In the case of a higher number of groups, a nonparametric Kruskall-Wallis ANOVA test was used instead. In order to evaluate the correlation between variables, Spearman’s rank correlation coefficient was used. *P* values of <0.05 were considered to be statistically significant. The entire statistical analysis was carried out using Statistica 10.0 software by Statsoft.

## Results

### Plasma Levels of Selected Extracellular microRNA

The normality of distribution of examined microRNA (miR-let-7a, miR-648a, miR-92a) was assessed using the Shapiro-Wilk test. The resulting *p* value (*p* < 0.05) allowed for the rejection of the null hypothesis, and nonparametric tests were used for subsequent analyses.

**Fig. 1:**
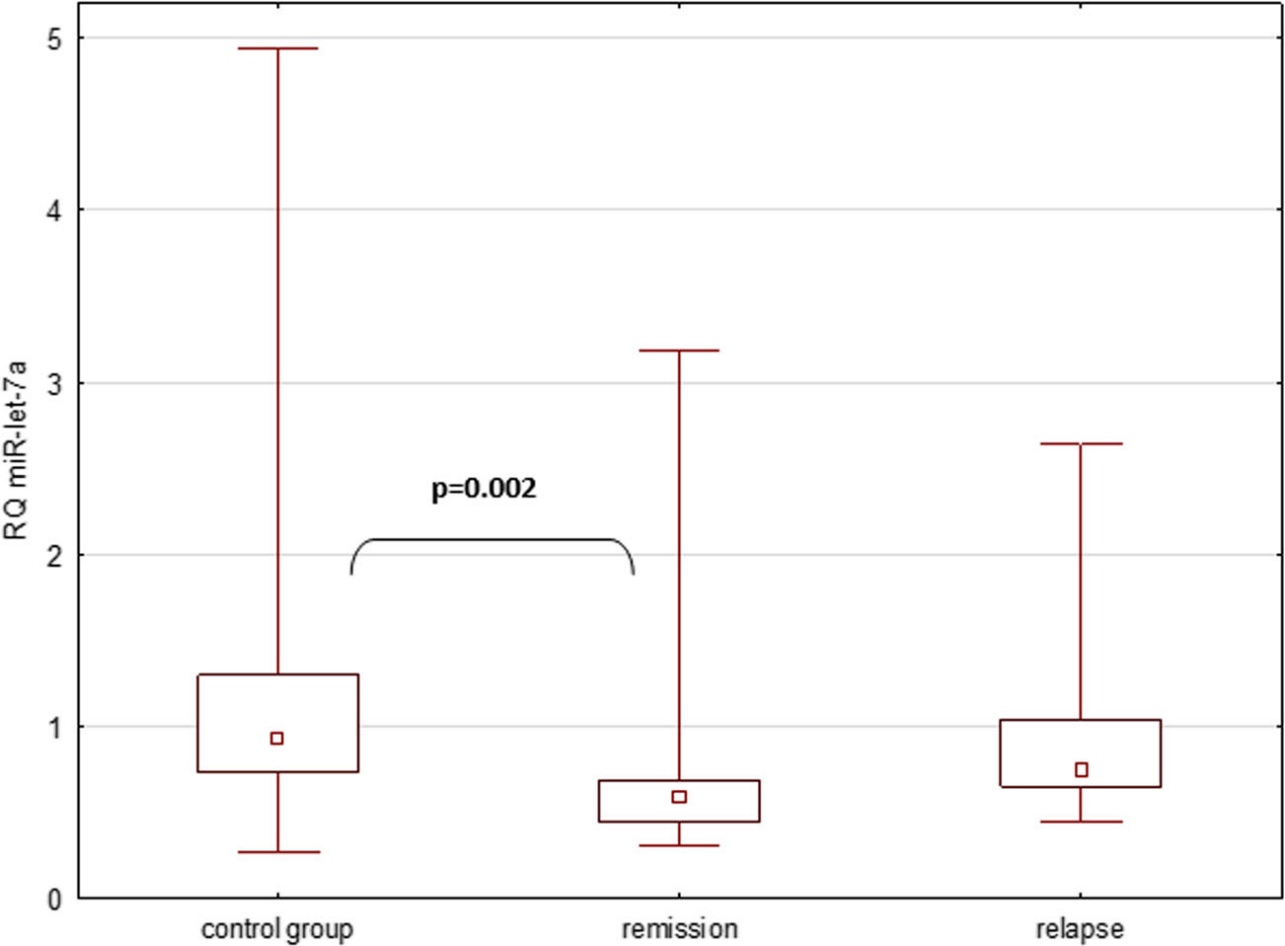
Expression of miR-let-7a in all analyzed groups, as described in Materials and Methods

There was a statistically significant difference in the expression of miR-let-7a among all groups included in the analysis (Kruskal-Wallis ANOVA *p* = 0.0035). A detailed analysis of relationships in subgroups revealed a statistically significant difference in expression levels of miR-let-7a—in patients in remission, the relative expression was lower than in the control group (Kruskal-Wallis ANOVA *p* = 0.002), whereas the remaining analyses were not statistically significant (Fig. 1).

**Fig. 2:**
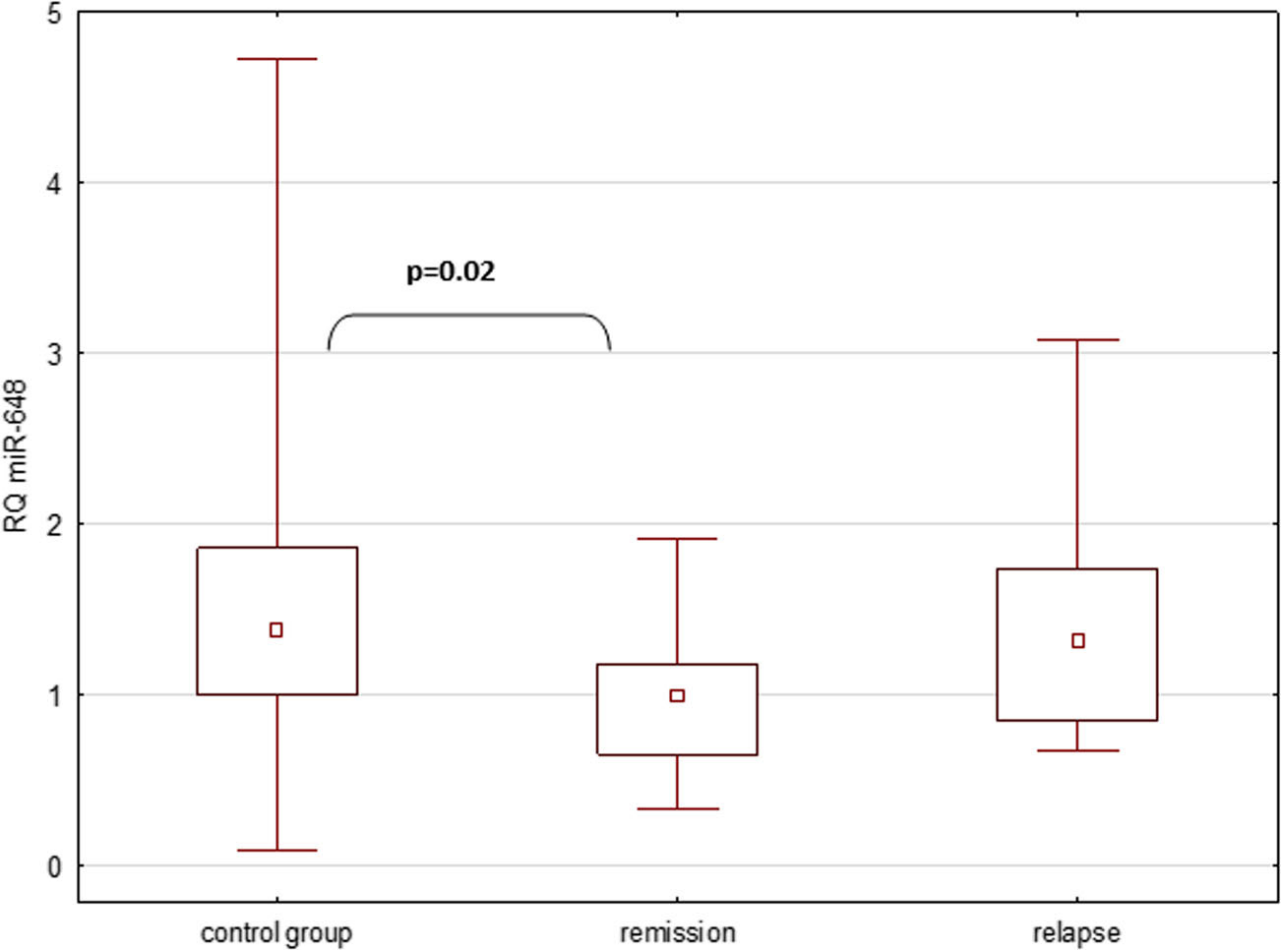
Expression of miR-648 in all analyzed groups, as described in Materials and Methods

The expression of miR-648a in all analyzed groups was also significantly different (Kruskal-Wallis ANOVA *p* = 0.024). The expression of miR-648a in patients in MS remission was lower than in the control group (Kruskal-Wallis ANOVA *p* = 0.02), whereas the remaining analyses were not statistically significant (Fig. 2).

The combined analysis of all three groups shows no significant difference in the expression of miR-92a (*p* = 0.19). Further analyses between subgroups showed no statistically significant differences (*p* = 0.24 for comparison between remission and control groups, *p* = 0.94 for comparison between relapse and control groups, and *p* = 0.43 for comparison between remission and relapse groups) (data not shown).

### Analysis of Correlation Between the Expression of Selected microRNA and Severity of the Disease

**Fig. 3:**
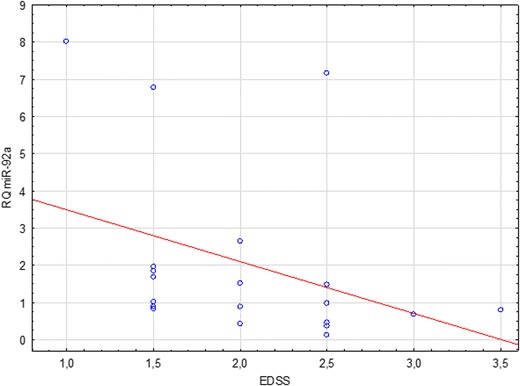
Correlation between miR-92a relative quantitation (RQ) and MS severity (EDSS scores) in patients with MS relapse

The presented study attempted to correlate relative levels of expression of selected miRNA with the severity of MS symptoms, as measured by the EDSS. Analyses were performed for relapse and remission subgroups, as well as the joint group of all patients (in relapse and remission). A statistically significant correlation was found between the relative expression of miR-92a and the EDSS in the relapse group. The observed result was a negative linear correlation (Spearman’s rank correlation, *p* = 0.01) (Fig. 3).

**Fig. 4:**
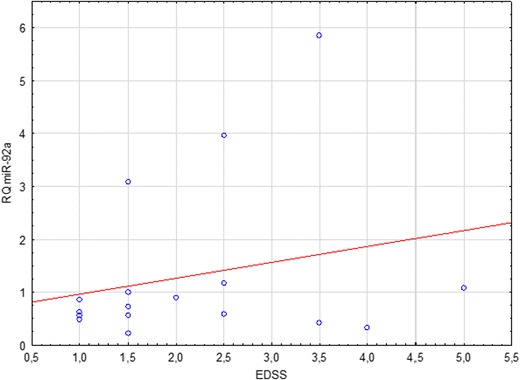
Correlation between miR-92a relative quantitation (RQ) and MS severity (EDSS score) in patients in MS remission

A positive linear correlation between the EDSS score and miR-92a expression in the remission group was also noted, although it was not statistically significant (Spearman’s rank correlation, *p* = 0.39) (Fig. 4).

**Fig. 5:**
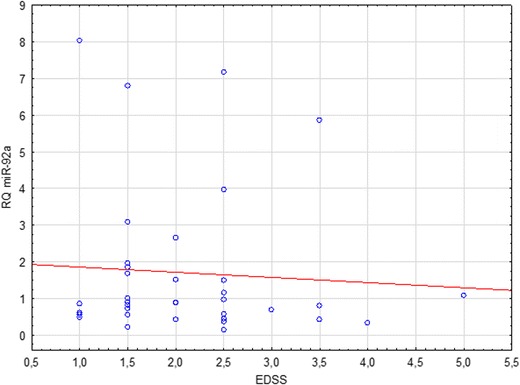
Correlation between miR-92a relative quantitation (RQ) and MS severity (EDSS score) in combined groups of MS patients (relapse + remission)

A negative linear correlation between the EDSS score and miR-92a expression in the joint group of patients (relapse and remission together) was noted. It was not statistically significant (Spearman’s rank correlation, *p* = 0.56) (Fig. 5).

### Analysis of Correlation Between the Expression of Selected microRNA and Cumulative Number of Relapses

**Fig. 6:**
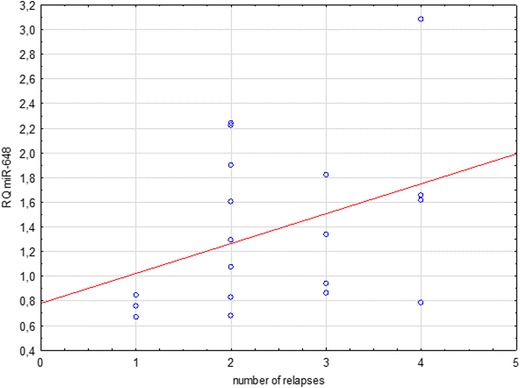
Correlation between miR-648a relative quantitation (RQ) and cumulative number of relapses in patients undergoing MS relapse

**Fig. 7:**
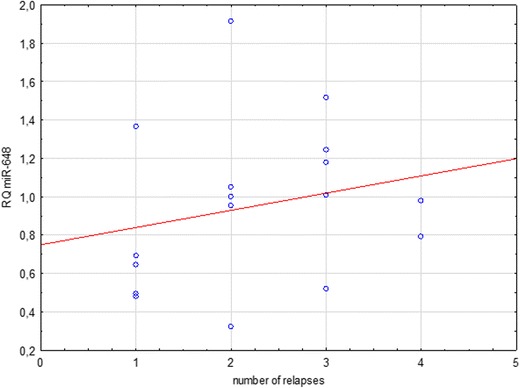
Correlation between miR-648a relative quantitation (RQ) and the number of relapses in MS patients in remission

**Fig. 8:**
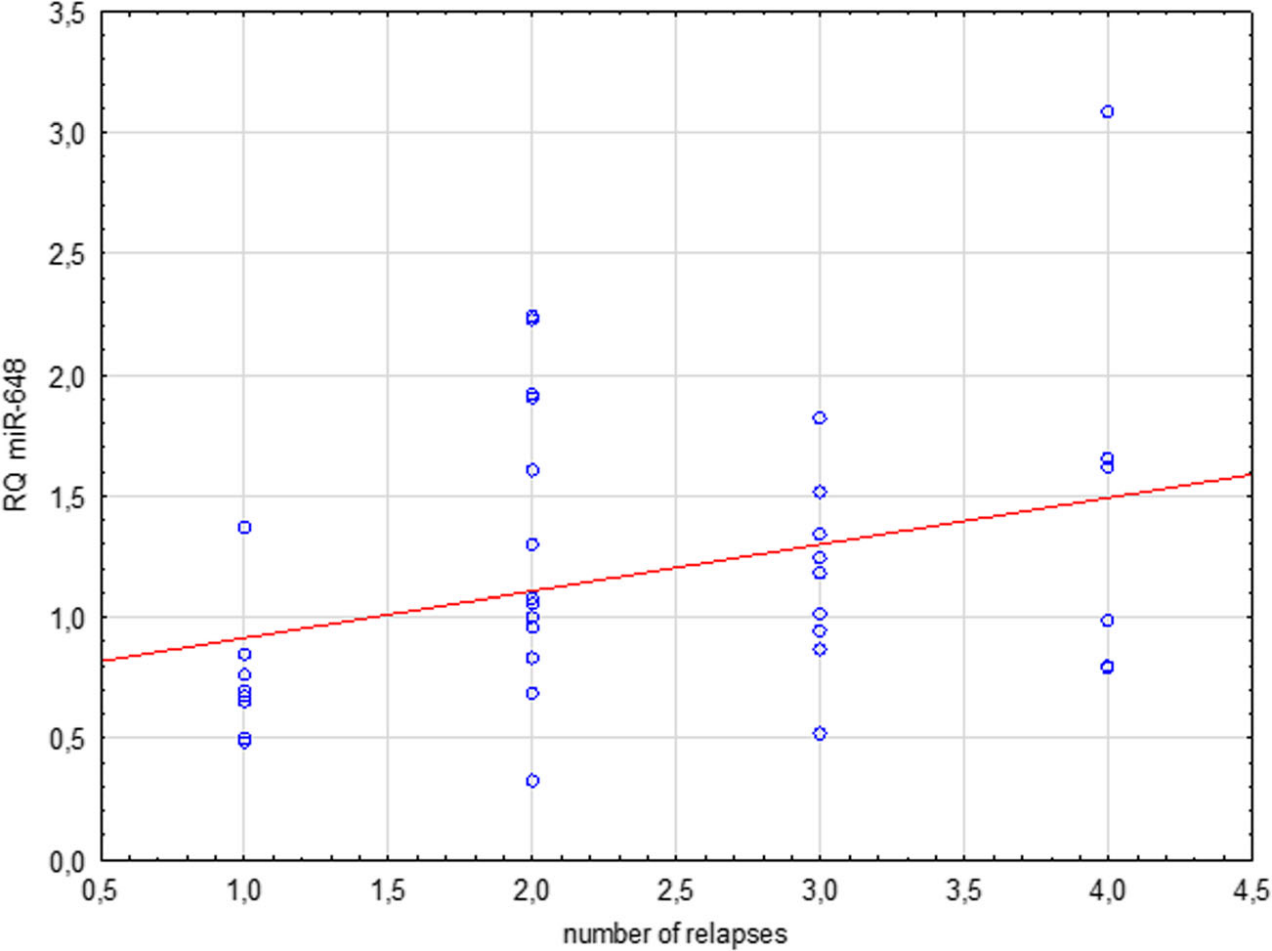
Correlation between miR-648a expression and cumulative number of relapses in combined group of all MS patients

Two distinct patterns have been observed upon the analysis of the number of flare-ups that patients have undergone and the expression of selected miRNA: lower miR-92a expression tended to correlate with more frequent flare-ups in the group of patients in remission (Spearman’s rank correlation, *p* = 0.1), while higher miR-648a expression tended to correlate with more frequent flare-ups in the group of patients in relapse (Spearman’s rank correlation, *p* = 0.09, Fig. 6). In the group of patients in remission, this tendency was also observed but it was weaker (Spearman’s rank correlation, *p* = 0.24; Fig. 7). The latter of the described correlations also holds true and is statistically significant when applied to the joint group of patients in remission and relapse (*p* = 0.03; Fig. 8).

### Analysis of Correlation Between WBC and Their Subpopulations (Lymphocytes, Neutrophils and Monocytes) and Relative Expression of Selected microRNA

All patients underwent a complete blood count with a WBC differential. Abnormalities of these lab results can stem from therapy itself or from coinciding pathologies, e.g., acute infections. An attempt to correlate WBC counts (in total as well as specific fractions) with relative expression of miRNA was made. No statistically significant correlations were found. The results are presented in Table 1.

**Table 1:** Comparison of total WBC counts (total and subpopulations) with relative expression of the studied miRNA

	miR-let-7a		miR-648a		miR-92a	
	Correlation rank	*p*	Correlation rank	*p*	Correlation rank	*p*
Patients in relapse						
WBC	−0.05	0.82	−0.20	0.42	−0.04	0.87
Neutrophils	−0.14	0.55	−0.31	0.18	−0.21	0.37
Lymphocytes	−0.04	0.86	0.03	0.89	0.35	0.13
Monocytes	−0.15	0.54	0.29	0.22	0.25	0.29
Patients in remission						
WBC	0.10	0.69	−0.004	0.99	0.14	0.58
Neutrophils	0.20	0.43	0.12	0.63	0.26	0.31
Lymphocytes	−0.11	0.68	−0.23	0.38	−0.07	0.80
Monocytes	−0.24	0.36	−0.19	0.48	−0.46	0.06
Patients in relapse and remission						
WBC	0.05	0.76	0.04	0.83	0.08	0.65
Neutrophils	0.03	0.86	−0.04	0.80	−0.07	0.83
Lymphocytes	0.02	0.91	0.10	0.56	0.24	0.16
Monocytes	−0.17	0.30	0.15	0.37	0.03	0.96

## Discussion

The search for an ideal marker of multiple sclerosis is still underway. As of yet, a molecule that would allow a quick and precise diagnosis, as well as the monitoring of the immune and pathological processes, has not been found. Currently, a group of endogenous, single-stranded noncoding RNA molecules, or microRNA (miRNA, miR), shows promise. It is the best known class of noncoding regulatory RNA to date. They are described as multifunctional molecules, as they are involved in both physiological as well as pathological processes. In the presented study, three selected extracellular microRNA molecules were investigated (miRNA-let-7a, miRNA-92a, and miRNA-648a) in patients in remission or undergoing a relapse of MS.

This study identified a member of the let-7 family, the hsa-let-7a, which helped differentiate the relapsing-remitting form of MS (RRMS) from the healthy control group (HC). In the case of miR-let-7a, there was a statistically significant difference in the expression across all of the groups involved in the study (*p* = 0.0035). A detailed analysis of the relationships in the subgroups revealed a significantly lower expression of miR-let-7a in the group of patients in remission compared to the control group (*p* = 0.002). In other cases, the differences were not statistically significant. In none of the studied groups, miR-let-7a correlates with patients’ EDSS scores; this could indicate that levels of miR-let-7a found in plasma are not a reliable marker of patients’ clinical condition. Previous studies focusing on extracellular miRNA-let-7a determined that these molecules were potent activators of Toll-like receptors (TLR) in macrophages and microglia (Roush and Slack 2008) and that they took part in cellular signaling, activation of stem cells, and neurogenesis (Swaminathan et al. 2012). Moreover, they were also involved in the regulation of the expression of anti-inflammatory cytokines, such as IL-10 and IL-13, in T cells in patients with MS (Kaushansky et al. 2010).

The analysis of miRNA-648a revealed a statistically significant difference in the expression between all the groups involved in the study (*p* = 0.024).A detailed analysis of the relationships in the subgroups revealed a significantly lower expression of miR-let-7a in the group of patients in remission compared to the control group (*p* = 0.02). miR-648a RQ comparisons between the remission and relapse groups (*p* = 0.12) as well as between the relapse and control groups (*p* = 0.67) proved to be insignificant. The study investigated the potential link between the relative expression of selected miRNA and the severity of MS symptoms, as measured by the EDSS score. In the case of miR-648a, no such correlation was observed.

Another observed tendency was for a higher expression of miR-648a in patients with a history of more frequent flare-ups within the relapse group (*p* = 0.09). This correlation is statistically significant in a joint analysis of patients in relapse and remission together (*p* = 0.03). The function of miR-648a is to modulate the expression of myelin-associated oligodendrocyte basic protein (MOBP) and the NR2C2 steroid nuclear receptor (Doi et al. 2008; Kaushansky et al. 2010). Changes in myelin-associated oligodendrocyte basic protein are seen in the pathogenesis of MS (Doi et al. 2008), while changes in NR2C2 affects the modulation of NR4A2. This is expressed in the T cells of MS individuals and acts as an essential transcription factor for triggering the production of inflammatory cytokines in MS (Doi et al. 2008; Siegel et al. 2012; Tsuchida et al. 2011). Increased expression of miRNA-648a in plasma might lead to decreased expression of MOBP, which in turn could be responsible for the reduction of durability and continuity of myelin sheaths in the CNS (Doi et al. 2008; Siegel et al. 2012; Tsuchida et al. 2011). It was therefore speculated that this miRNA targets FOXD1, a member of the forkhead homeobox/winged helix family of transcription factors (Tsuchida et al. 2011).

No statistically significant differences in the expression across all studied groups were found concerning the third analyzed miRNA—the miR-92a (*p* = 0.19). Similarly, comparisons of its relative expression in relapse vs. remission group (*p* = 0.43), remission vs. control group (*p* = 0.24), and relapse vs. control group (*p* = 0.94) proved insignificant. Detailed analyses of correlation were carried out in all subgroups, including a joint group of patients in remission and in relapse together. The only statistically significant observation was a negative linear correlation between miR-92a RQ and the EDSS score in the group of patients undergoing an MS relapse (*p* = 0.01).

An attempt to correlate leukocyte counts (total and subpopulations) with relative expression levels of the studied miRNA was made (Table 1). No statistically significant results were obtained, which may signify that changes in WBC counts (which could be attributed to the MS therapy as well) did not influence the observed expression levels of the studied miRNA.

Previous studies demonstrate that miR-92 targets CD40 signaling pathways, which were found to be upregulated in RRMS disease. Those targets are involved in cell cycle regulation and belong to the miR-17-92 miRNA cluster (Balashov et al. 1997; Gandhi et al. 2013; Juntilla and Koretzky 2008; Xiao et al. 2008). Differential expression of the miR-17-92 cluster had previously been reported in relation to cancers and in multiple sclerosis, and it is involved in the modulation of the proliferation and activation of naive CD4+ T cells. It had also been identified as a contributing factor in the development of lymphoproliferative diseases in a murine model of autoimmunity, where raised counts of CD4+ T cells with an activated phenotype were noted (Balashov et al. 1997; Gandhi et al. 2013; Juntilla and Koretzky 2008; Xiao et al. 2008). Reduced expression of miR-92a has been observed in B cells of individuals with MS. A pathway potentially regulated by the miR-17-92 cluster is the PI3K/Akt pathway, known to regulate different stages of lymphocyte development, activation, and survival (Fenoglio et al. 2012; Guerau-de-Arellano et al. 2012; Reid et al. 2011).

miRNA molecules are of growing importance in many research areas. A thorough understanding of microRNA profiles in a given disease may aid in a more quick and accurate diagnosis. These molecules also appear to be universal prognostic factors. Expression profiles of particular miRNA characterize selected types and stages of MS with a high degree of specificity. The biological material necessary for such testing is easy to obtain, since miRNA expression can be evaluated based on plasma concentrations. This increases the availability of research material and makes analyses easier to perform.

The obtained results suggest that extracellular miRNA are interesting and promising candidates for biomarkers of multiple sclerosis (MS), although further research is necessary. As had been demonstrated in previous studies, miRNA expression differs depending on the stage of the disease (relapse vs. remission), which allows for the inference that these molecules could aid in the early diagnosis of the disease. The next essential step is a larger study on a broader group of patients, representative of various clinical patterns of MS and other parameters. If those efforts lead to the discovery of miRNA molecules with high enough specificity, further studies will be necessary to evaluate their expression not only in MS but also in other immune-related and neurodegenerative diseases—for example, Parkinson’s disease, where similar studies are also being conducted (Heman-Ackah et al. 2013). Only then would it be possible to establish how specific and in turn how useful in clinical practice those new markers could really be.
